# Evidence of niche shift and invasion potential of *Lithobates catesbeianus* in the habitat of Mexican endemic frogs

**DOI:** 10.1371/journal.pone.0185086

**Published:** 2017-09-27

**Authors:** Jorge Luis Becerra López, Citlalli Edith Esparza Estrada, Ulises Romero Méndez, José Jesús Sigala Rodríguez, Irene Goyenechea Mayer Goyenechea, Jesús Martín Castillo Cerón

**Affiliations:** 1 Centro de Investigación en Sustentabilidad Energética y Ambiental del Noreste, Universidad Autónoma del Noreste, Tamaulipas, México; 2 Centro de Investigaciones Biológicas, Laboratorio de Sistemática Molecular, Universidad Autónoma del Estado de Hidalgo, Hidalgo, México; 3 Laboratorio de Sistemas de Información Geográfica, Facultad de Ciencias Biológicas, Universidad Juárez del Estado de Durango, Durango, México; 4 Colección Zoológica de la Universidad Autónoma de Aguascalientes, Aguascalientes, México; 5 Museo de Paleontología, Universidad Autónoma del Estado de Hidalgo, Hidalgo, México; Universitat Trier, GERMANY

## Abstract

Invasive alien species are one of most severe threats to biodiversity and natural resources. These biological invasions have been studied from the niche conservatism and niche shifts perspective. Niche differentiation may result from changes in fundamental niche or realized niche or both; in biological invasions, niche differences between native and non-native ranges can appear through niche expansion, niche unfilling and niche stability. The American bullfrog *Lithobates catesbeianus* is an invasive species that can have negative impacts on native amphibian populations. This research examines the climate niche shifts of this frog, its potential range of expansion in Mexico and the risk of invasion by bullfrog in the habitats of 82 frog species endemic to Mexico, that based on their climatic niche similarity were divided in four ecological groups. The results indicate that species in two ecological groups were the most vulnerable to invasion by bullfrog. However, the climate niche shifts of *L*. *catesbeianus* may allow it to adapt to new environmental conditions, so species from the two remaining groups cannot be dismissed as not vulnerable. This information is valuable for decision making in prioritizing areas for conservation of Mexican endemic frogs.

## Introduction

Invasive alien species are one of most severe threat to biodiversity and natural resources [1], which have been shown to affect ecosystem services and decrease native species abundance through mechanisms such as predation, hybridization, competition and indirect effects [2]. The establishment success and subsequent distribution of an invasive species is finally determined by the species association to abiotic variables such as climate. These associations can be interpreted through the concept of niche [3]. It is already known that a niche is the set of biotic and abiotic conditions in which a species is able to persist and maintain stable population sizes [4]. Hutchinson distinguished between the fundamental environmental niche (i.e. genetically and physiologically determined) and the realized environmental niche (i.e. constraints arising from interspecific competition) [5].

Biological invasions have been studied from a niche conservatism perspective (the tendency of species to retain ancestral ecological characteristics [6]) and niche shift perspective (any change in the position of either the fundamental or realized niche of a species) or both [5]. Niche conservatism and niche shifts can have important implications for understanding speciation, effects of climate change and biological invasions [6, 7]. In this way, niche differentiation may result from changes in either the fundamental niche or the realized niche of the species [7]. Some studies of invasion provided evidence suggesting that the climatic niche occupied by species may not be conserved between their native and invaded ranges, as documented by observed niche shifts for plants [7–9], insects [10–11], fish [12] and amphibians [13].

In the case of biological invasions, niche differences between native and non-native ranges can appear through three different processes: 1) niche expansion (i.e. species colonizing novel environmental conditions relative to their native range), 2) niche unfilling (i.e. a partial filling of the native niche in the invaded range [14]) and 3) niche stability (i.e. proportion of the exotic niche overlapping with the native niche [5]) [15–16]. Evaluating whether these processes lead to significant realized niche differentiation between native and non-native ranges entails testing two different hypotheses: 1) niche equivalency (native and non-native niches are indistinguishable) and 2) niche similarity (whether niches are more similar than expected by chance [17]) [16].

*Lithobates catesbeianus* is an invasive species whose native range is in central and eastern United States [18]. It has been introduced in more than 40 countries in four continents, for commercial purposes [19–20]; the earliest free-ranging populations were recorded in various states of Mexico; Nuevo Leon in 1853, Tamaulipas in 1898, Sinaloa in 1969 [21–23], and more recent records in different areas of the country [24]. Bullfrogs can have negative impacts on native amphibian populations; the large tadpoles of this species can outcompete the larvae of native species; moreover, adults are generalist predators and also prey on other amphibians [20]. Furthermore, introduced bullfrogs can be carriers of *Batrachochytrium dendrobatidis*, a fungus that is the agent of chytridiomycosis, an emerging infectious disease that is considered one of the central causes of global amphibian decline and extinctions [25]. In Mexico, bullfrogs have been reported as opportunistic predators and competitors of endemic amphibians in the areas they have invaded [26], affecting some species in risk categories [23]. In addition to negatively impacting native species and natural ecosystems, bullfrogs represent a threat to the biodiversity of Mexico [27] where about half of the amphibian species are endemic to the country [28].

Bearing in mind that native species with climatic preferences similar to the invasive species may be considered vulnerable [29–30] and that invasive species can undergo a niche shift in new environmental conditions and adapt quickly to colonized new areas [5, 7, 31], in the present study, we examined the shift in the realized niche of this frog, its potential range of expansion in Mexico and the risk of invasion by bullfrog in the habitats of 82 frog species endemic to Mexico, based on the climatic niche similarity.

## Materials and methods

### Presence data

**Fig 1:**
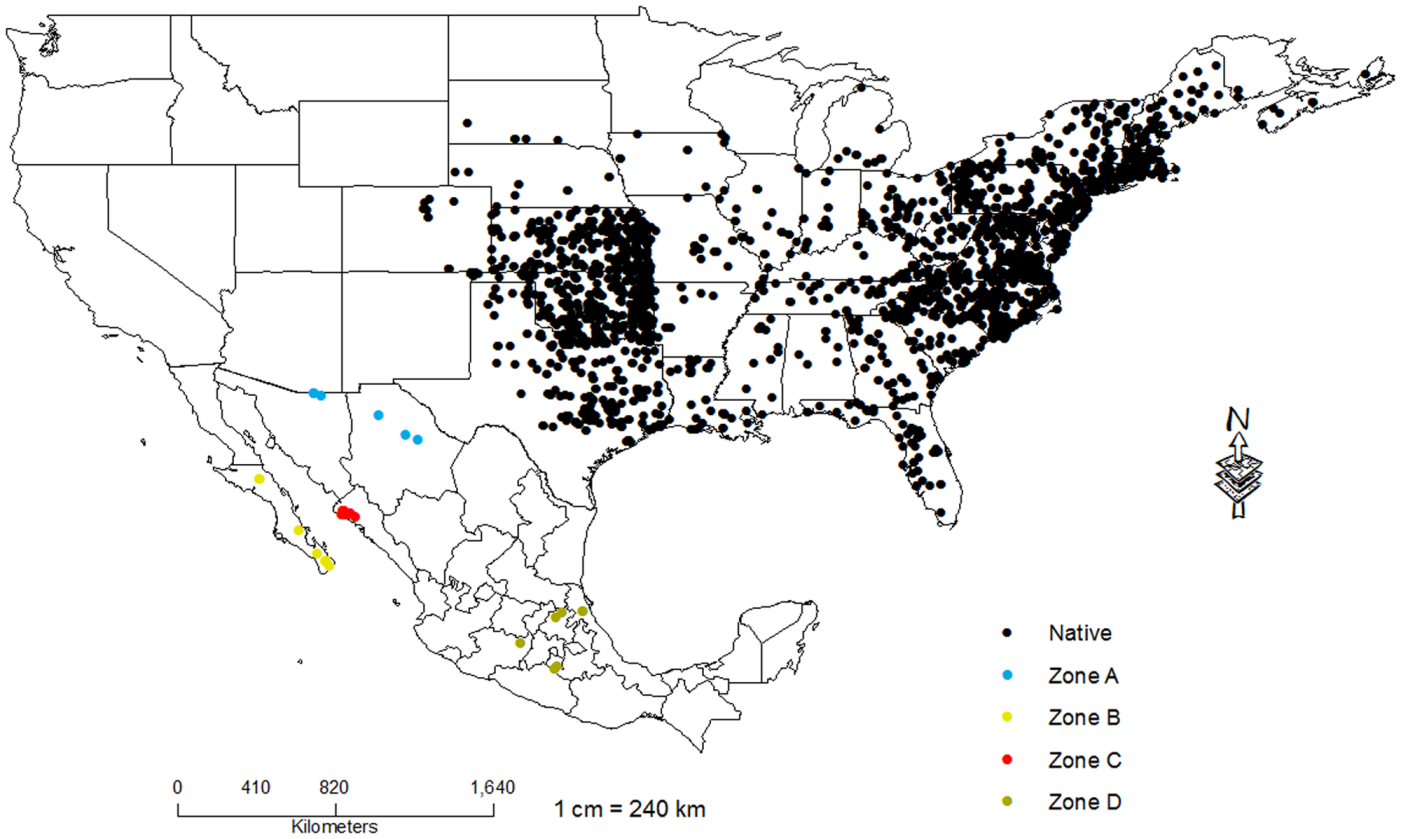
Distribution of *Lithobates catesbeianus* native and invaded ranges. The black dots indicate the native distribution of *L*. *catesbeianus* in the United States. The dots in yellow, blue, red and green represent the invaded distribution of bullfrogs in Mexico.

Occurrence data were compiled from Global Biodiversity Information Facility database (GBIF, http://www.gbif.org), for *L*. *catesbeianus*, yielding 6322 presence records from the native range in the United States and from invaded range in Mexico (Fig 1). Govindarajulu et. al. [32] listed the preferred optimum environmental temperature of bullfrogs in native range to be 15–32°C. While, Wright and Wright [33] listed shoreline cover as an important habitat for adults and shallow water as important habitat for tadpoles and Dickerson [34] suggested that bullfrogs prefer deep water with shallow margins and a cover of submerged and emergent vegetation.

Presence records from invaded range in Mexico were divided in four zones: 1) Zone A (Sonora and Chihuahua) where vegetation is xerophytic scrub and grassland [35], mean annual precipitation varies from 175 to 400 mm and mean annual temperature is 18–22°C [36]; 2) Zone B (Baja California Sur) where vegetation is xerophytic scrub [35], mean annual precipitation is less than 200 mm and mean annual temperature fluctuates between 18–22°C [36]; 3) Zone C (Sinaloa) where vegetation is thorn lowland forest and wetland [35], precipitation varies from less than 700 mm and mean annual temperature is 22–26°C [36]; and 4) Zone D (Veracruz, Hidalgo, Michoacán and Morelos) where vegetation is deciduous forest, pine-oak forest, mountain mesophilic forest, medium sub-evergreen forest moreover, medium and high evergreen forest [35], mean annual temperature and precipitation varies for Veracruz from 24–26°C and 1 400–1 600 mm; Hidalgo from 12–22°C. and 900–1 600 mm; Michoacán from 12–22°C and 800–1 300 mm and Morelos from 20–26°C and 800–1 100 mm [36].

We defined native and exotic ranges of the bullfrog based on previous literature [37–39]. In addition, we compiled occurrence data for 82 endemic anurans of Mexico. Records outside the known distribution of species were removed from the database. The number of records per species was variable, but every species had at least five presence points (S1 Table).

### Climatic variables

Climate information was obtained from the 19 layers in the current climate available in WorldClim database version 1.4 [40]. These layers contain climatic averages of weather conditions recorded from 1950–2000 with a spatial resolution of 30 arc- seconds (~ 1 km). The right choice of climatic variables based on the biology of the species under study plays an important role for robust modeling [41], on the understanding that the climate sets the broad contours of the species distribution [42].

Environmental variables were selected using the ArcMap 10.1 software, where 10,000 geographic points were randomly generated within the range of American Bullfrog in North America (The United States and Mexico). The addition of 10,000 random points was based on the criteria of not discriminating (non-repetitive) relevant information, but segregated geographical areas within the range of the bullfrog climate information [43], Then, information of the 19 environmental variables from the current climate was added to these points [44]. With the generated information, a bivariate correlation analysis was conducted in order to reduce the multicollinearity between the input variables [45]. Predictor variables that were highly correlated when (| rs | ≥ 0.7) were excluded. The following six climatic variables were retained: annual mean temperature (bio1), monthly mean diurnal range (bio2), isothermality (bio3), mean temperature of wettest quarter (bio8), annual precipitation (bio12), and precipitation seasonality (bio15).

### Climatic niche overlap

We used the principal component analysis (PCA) approach proposed by Broennimann et al. [46] to measure equivalence and similarity between the realized niche of the bullfrog between the native range and the Mexican invaded range (with the six variables retained in bivariate correlation analysis). This method compares the environmental conditions available for a species within a defined study extent (background) with its observed occurrences and calculates the available environmental space defined by the first two axes from the PCA. This method corrects for sampling bias using a smooth kernel density function [46].

The niche overlap between native and invaded ranges was calculated using Schoener’s D metric [47], which varies from 0 (no overlap between niches) to 1 (whole overlap). Niche equivalency and similarity test were built from the methodology described in Broennimann et al. [46]. The niche equivalency test determines whether niches of two entities in two geographical ranges are equivalent (i.e. whether the niche overlap is constant when randomly reallocating the occurrences of both entities among the two ranges). All occurrences are pooled and randomly split into two datasets, maintaining the number of occurrences as in the original datasets. This process is repeated 100 times (to ensure that the null hypothesis can be rejected with high confidence). If the observed value of D falls within the density of 95% of the simulated values, the null hypothesis of niche equivalency cannot be rejected. Therefore, the niche similarity test differs from the equivalency test because the former examines whether the overlap between observed niches in two ranges is different from the overlap between the observed niche in one range and niches selected at random from the other range. The test of niche similarity is also based on 100 repetitions. If the observed overlap is greater than 95% of the simulated values, the entity occupies environments in both of its ranges that are more similar to each other than expected.

We also measured the proportion of the invaded niche of *L*. *catesbeianus* that was stable (i.e. the invaded niche overlapping with the native niche), unfilled (i.e. the native niche non-overlapping with the invaded niche) and expanding (i.e. the invaded niche non-overlapping with the native niche) compared to its native niche [14–15]. Finally, we used the niche overlap test to compare equivalence and similarity between the realized niche of *L*. *catesbeianus* from the invaded range in Mexico and the realized niche of 82 endemic anurans of Mexico (S1 Table). All the analyses were done in the statistical software R3.1.3 [48].

### Climatic niche modeling

Maximum entropy model (MaxEnt, version 3.3.3k [49]) was used to represent potential distribution of *L*. *catesbeianus*. The MaxEnt model was chosen because it uses presence-background data (i.e., randomly selected absences from areas that have been accessible to the species), it generally has a better performance for invasive species than other niche modeling algorithms. In the case of presence-absence models, absence data may not be reliable; a species may go undetected or it may not have had sufficient time to disperse to new locations yet [50].

MaxEnt predict habitat suitability as a function of environmental variables and species occurrence data. This habitat suitability is represented by a scale ranging from 0 (low suitability) to one (high suitability) [50–53]). Proper calibration and evaluation was necessary to reduce the complexity of the model [54] considering the choice of: i) accessible area (background or M area); ii) the type of variables that Maxent constructs (features) and iii) the type of model output (raw, cumulative, logistic), as these considerations affect the inferences to be made [44]. Proper calibration and evaluation is also especially important for data sets suffering from sampling bias, and for studies that require transfer models through space or time [55–56].

In this study, the calibration and evaluation method for *L*. *catesbeianus* modeling was carried out using the library "ENMeval" [57] in the statistical software R 3.1.3 [48] considering the known distribution of this species in the native range in the United States and invaded ranges in Mexico, the climatic variables defined above and elevation data. The calibrated model was evaluated by calculating the coefficient standardized Akaike information criterion (AICc). The AICc, provides information on the relative quality of a model [54]. Because the AICc is calculated using the data set it is not affected by the method chosen for the data partition [57]. The model with the lowest AICc was selected as the best fit for the species.

The information obtained from the calibrated model was projected to Mexico and the United States, considering the environmental variables described above to the current climate and using the Maxent software [49]. The number of repetitions was 100 replicas [58], obtaining a model of ecological niche geographically represented as a map of habitat suitability under current climatic conditions for *L*. *catesbeianus*.

## Results

### Overlap climatic niche

**Fig 2:**
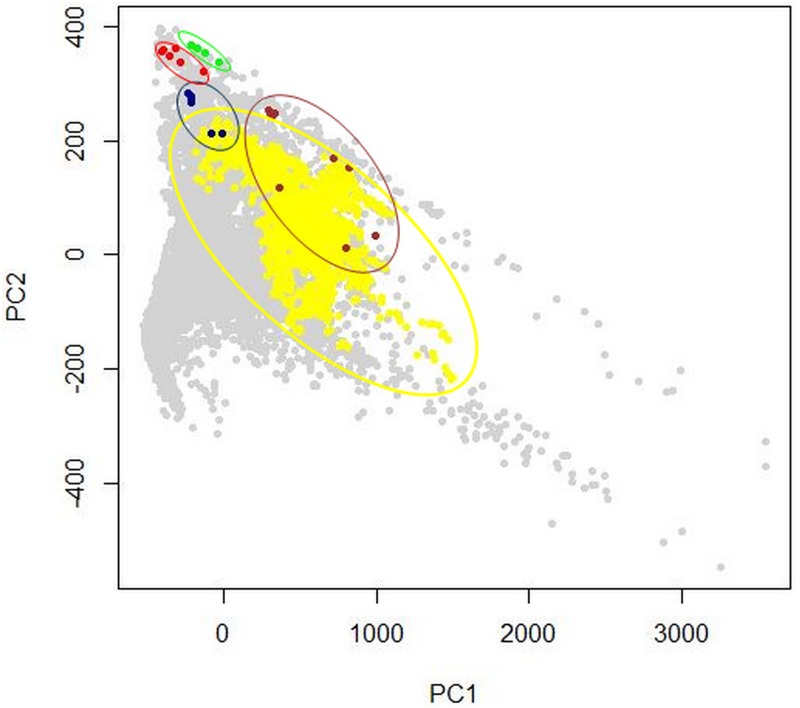
Principal component analysis. The gray dots represent the multivariate climatic space of the distribution of *L*. *catesbeianus* in the United States and Mexico. The yellow dots indicate the distribution of bullfrogs in climate space of native range, dark blue dots shows the distribution of this frog in climate space of Zone A (Sonora and Chihuahua), red dots in Zone B (Baja California Sur), green dots in Zone C (Sinaloa) and brown dots in Zone D (Veracruz, Hidalgo, Michoacán and Morelos).

Principal component analysis showed niche overlap between the climatic space in the native range of *L*. *catesbeianus* and the climatic space of the invaded zones A and D in Mexico (Fig 2). In both comparisons, the first axis explains the greatest of variations in the environmental conditions for Zone A with 74.84% and Zone D with 55.22% (S1 and S2 Figs).

The equivalence test of the native and invaded realized niche of *L*. *catesbeianus* was significant (P<0.05). The analysis of similarity (native vs invaded and invaded vs native) showed that the realized niche in the native range and in the invaded zones A and D were significantly more similar than expected, but invaded zones A and D were not significant compared to native range. The similarity analyses between the native range and the invaded zones B and C were not significant in either direction (native vs invaded and invaded vs native) (Table 1).

**Table 1:** Equivalence and similarity.

	Equivalence	Similarity		Expansion	Stability	Unfilling
		X→Z	Z→X			
**Zone A**	D = 0.0003	D = 0.003–0.003		0.340	0.659	0.990
	P = 0.01	P = 0.01–0.68				
**Zone B**	D = 0	D = 0–0		0.944	0.055	0.996
	P = 0.01	P = 1–1				
**Zone C**	D = 0	D = 0–0		1	0	1
	P = 0.01	P = 1–1				
**Zone D**	D = 0.06	D = 0.06–0.06		0.532	0.467	0.174
	P = 0.01	P = 0.03–0.3				

(X → Z: from native to exotic; Z → X: from exotic to native) are measured in terms of niche overlap (Schoener’s *D*) and P means statistical significance. Expansion and Stability are proportions of the non-overlapping and overlapping exotic niche, respectively, with the native niche. Unfilling is the proportion of the native niche available, but non-occupied in the exotic niche.

The equivalence test between the realized niche of *L*. *catesbeianus* in Mexico and the realized niche for each of the 82 species of frogs was significant (P<0.05). We classified the 82 species of frogs into four groups according to the results of similarity test (Table 2). Group 1 includes those species where significant differences in both directions, in Group 2 only *L*. *catesbeianus* present significant differences and Group 3 in the opposite direction. Finally, Group 4 does not present significant differences in either direction (Table 2).

**Table 2:** Equivalence and similarity analysis of *L*. *catesbeianus* vs. endemic frogs.

**Group 1 (N = 22)**			
**Species**	**Equivalence**	**Similarity X→Z Z→X**	
*Charadrahyla chaneque* (Duellman, 1961)	D = 0.19 P = 0.01	D = 0.19–0.19	P = 0.009–0.03
*Charadrahyla nephila* (Mendelson and Campbell, 1999)	D = 0.04 P = 0.01	D = 0.04–0.04	P = 0.01–0.04
*Craugastor occidentalis* (Taylor, 1941)	D = 0.01 P = 0.01	D = 0.34–0.34	P = 0.009–0.02
*Craugastor omiltemanus* (Günther, 1900)	D = 0.25 P = 0.01	D = 0.25–0.25	P = 0.009–0.01
*Craugastor vocalis* (Taylor, 1940)	D = 0.26 P = 0.01	D = 0.26–0.26	P = 0.009–0.009
*Dendropsophus sartori* (Fitzinger, 1843)	D = 0.16 P = 0.01	D = 0.16–0.16	P = 0.02–0.03
*Diaglena spatulata* (Günther, 1882)	D = 0.02 P = 0.01	D = 0.02–0.02	P = 0.009–0.009
*Duellmanohyla ignicolor* (Duellman, 1961)	D = 0.08 P = 0.01	D = 0.08–0.08	P = 0.009–0.009
*Ecnomiohyla miotympanum* (Cope, 1863)	D = 0.21 P = 0.01	D = 0.21–0.21	P = 0.009–0.06
*Eleutherodactylus longipes* (Baird, 1859)	D = 0.32 P = 0.01	D = 0.32–0.32	P = 0.009–0.03
*Eleutherodactylus teretistes* (Duellman, 1958)	D = 0.13 P = 0.01	D = 0.13–0.13	P = 0.009–0.06
*Exerodonta chimalapa* (Mendelson and Campbell, 1994)	D = 0.14 P = 0.01	D = 0.14–0.14	P = 0.009–0.009
*Incilius marmoreus* (Wiegmann, 1833)	D = 0.36 P = 0.01	D = 0.36–0.36	P = 0.009–0.009
*Incilius mazatlanensis* (Taylor, 1940)	D = 0.47 P = 0.01	D = 0.47–0.47	P = 0.009–0.009
*Incilius spiculatus* (Mendelson, 1997)	D = 0.05 P = 0.01	D = 0.05–0.05	P = 0.009–0.01
*Pachymedusa dacnicolor* (Cope, 1864)	D = 0.37 P = 0.01	D = 0.37–0.37	P = 0.009–0.009
*Plectrohyla sabrina* (Caldwell, 1974)	D = 0.001 P = 0.01	D = 0.001–0.001	P = 0.01–0.06
*Plectrohyla siopela* (Duellman, 1968)	D = 0.03 P = 0.01	D = 0.03–0.03	P = 0.009–0.05
*Plectrohyla lacertosa* (Bumzahem and Smith, 1954)	D = 0.07 P = 0.01	D = 0.07–0.07	P = 0.009–0.02
*Lithobates magnaocularis* (Frost and Bagnara, 1974)	D = 0.33 P = 0.01	D = 0.33–0.33	P = 0.009–0.009
*Lithobates pustulosus* (Boulenger, 1883)	D = 0.32 P = 0.01	D = 0.32–0.32	P = 0.009–0.01
*Tlalocohyla smithii* (Boulenger, 1902)	D = 0.35 P = 0.01	D = 0.35–0.35	P = 0.009–0.009
**Group 2 (N = 31)**			
**Species**	**Equivalence**	**Similarity X→Z Z→X**	
*Anaxyrus compactilis* (Wiegmann, 1833)	D = 0.16 P = 0.01	D = 0.17–0.17	P = 0.009–0.81
*Anaxyrus kelloggi* (Taylor, 1938)	D = 0.12 P = 0.01	D = 0.12–0.12	P = 0.009–0.15
*Anaxyrus mexicanus* (Brocchi, 1879)	D = 0.03 P = 0.01	D = 0.03–0.03	P = 0.02–0.88
*Charadrahyla altipotens* (Duellman, 1968)	D = 0.01 P = 0.01	D = 0.01–0.01	P = 0.009–0.54
*Craugastor pozo* (Johnson and Savage, 1995)	D = 0.02 P = 0.01	D = 0.02–0.02	P = 0.009–0.21
*Craugastor tarahumaraensis* (Taylor, 1940)	D = 0.13 P = 0.01	D = 0.13–0.13	P = 0.009–0.89
*Eleutherodactylus albolabris* (Taylor 1943)	D = 0.11 P = 0.01	D = 0.11–0.11	P = 0.009–0.79
*Eleutherodactylus dilatus* (Davis and Dixon, 1955)	D = 0.07 P = 0.01	D = 0.07–0.07	P = 0.009–0.45
*Eleutherodactylus modestus* (Taylor, 1942)	D = 0.03 P = 0.01	D = 0.03–0.03	P = 0.01–0.95
*Eleutherodactylus nitidus* (Peters, 1870)	D = 0.23 P = 0.01	D = 0.23–0.23	P = 0.009–0.15
*Eleutherodactylus nivicolimae* (Dixon and Webb, 1966)	D = 0.02 P = 0.01	D = 0.02–0.02	P = 0.009–0.8
*Hyla plicata* (Brocchi, 1877)	D = 0.09 P = 0.01	D = 0.09–0.09	P = 0.009–0.51
*Incilius cavifrons* (Firschein, 1950)	D = 0.01 P = 0.01	D = 0.01–0.01	P = 0.009–0.95
*Incilius occidentalis* (Camerano, 1879)	D = 0.25 P = 0.01	D = 0.25–0.25	P = 0.009–0.21
*Incilius perplexus* (Taylor, 1943)	D = 0.15 P = 0.01	D = 0.15–0.15	P = 0.04–0.12
*Megastomatohyla pellita* (Duellman, 1968)	D = 0.04 P = 0.01	D = 0.04–0.04	P = 0.01–0.26
*Plectrohyla cembra* (Caldwell, 1974)	D = 0.07 P = 0.01	D = 0.07–0.07	P = 0.009–0.4
*Plectrohyla charadricola* (Duellman, 1964)	D = 0.02 P = 0.01	D = 0.02–0.02	P = 0.009–0.68
*Plectrohyla mykter* (Adler and Dennis, 1972)	D = 0.03 P = 0.01	D = 0.03–0.03	P = 0.009–0.48
*Plectrohyla crassa* (Brocchi, 1877)	D = 0.07 P = 0.01	D = 0.07–0.07	P = 0.009–0.48
*Plectrohyla cyclada* (Campbell and Duellman, 2000)	D = 0.08 P = 0.01	D = 0.08–0.08	P = 0.01–0.17
*Plectrohyla robertsorum* (Taylor, 1940)	D = 0.03 P = 0.01	D = 0.03–0.03	P = 0.009–0.75
*Lithobates berlandieri* (Baird, 1859)	D = 0.001 P = 0.01	D = 0.001–0.001	P = 0.01–0.07
*Lithobates megapoda* (Taylor, 1942)	D = 0.08 P = 0.01	D = 0.08–0.08	P = 0.01–0.46
*Lithobates neovolcanicus* (Hillis and Frost, 1985)	D = 0.03 P = 0.01	D = 0.03–0.03	P = 0.009–0.83
*Lithobates johni* (Blair, 1965)	D = 0.03 P = 0.01	D = 0.03–0.03	P = 0.05–0.71
*Lithobates omiltemanus* (Günther, 1900)	D = 0.06 P = 0.01	D = 0.06–0.06	P = 0.009–0.29
*Lithobates spectabilis* (Hillis and Frost, 1985)	D = 0.2 P = 0.01	D = 0.2–0.06	P = 0.009–0.41
*Lithobates tlaloci* (Hillis and Frost, 1985)	D = 0.00008P = 0.01	D = 0.00008–0.00008	P = 0.009–0.48
*Lithobates zweifeli* (Hillis, Frost, and Webb, 1984)	D = 0.17 P = 0.01	D = 0.17–0.17	P = 0.01–0.13
*Smilisca dentata* (Smith, 1957)	D = 0.06 P = 0.01	D = 0.06–0.06	P = 0.009–0.71
**Group 3 (N = 11)**			
**Species**	**Equivalence**	**Similarity X→Z Z→X**	
*Craugastor berkenbuschii* (Peters, 1870)	D = 0.16 P = 0.01	D = 0.16–0.16	P = 0.54–0.02
*Craugastor hobartsmithi* (Taylor, 1937)	D = 0.12 P = 0.01	D = 0.12–0.12	P = 0.75–0.009
*Craugastor mexicanus* (Brocchi, 1877)	D = 0.13 P = 0.01	D = 0.13–0.13	P = 0.44–003
*Craugastor montanusi* (Lynch, 1965)	D = 0.05 P = 0.01	D = 0.05–0.05	P = 0.67–0.02
*Craugastor rugulosus* (Cope, 1870)	D = 0.06 P = 0.01	D = 0.06–0.06	P = 0.66–0.04
*Exerodonta pinorum* (Taylor, 1937)	D = 0.05 P = 0.01	D = 0.05–0.05	P = 0.49–0.009
*Plectrohyla bistincta* (Cope, 1877)	D = 0.11 P = 0.01	D = 0.11–0.11	P = 0.89–0.009
*Plectrohyla cyanomma* (Caldwell, 1974)	D = 0.8 P = 0.01	D = 0.08–0.08	P = 0.35–0.05
*Ptychohyla erythromma* (Taylor, 1937)	D = 0.05 P = 0.01	D = 0.05–0.05	P = 0.38–0.009
*Lithobates sierramadrensis* (Taylor, 1939)	D = 0.12 P = 0.01	D = 0.12–0.12	P = 0.6–0.05
*Tlalocohyla godmani* (Günther, 1901)	D = 0.16 P = 0.01	D = 0.16–0.16	P = 0.18–0.009
**Group 4 (N = 18)**			
**Species**	**Equivalence**	**Similarity X→Z Z→X**	
*Bromeliohyla dendroscarta* (Taylor, 1940)	D = 0.11 P = 0.01	D = 0.11–0.11	P = 0.18–0.26
*Charadrahyla taeniopus* (Günther, 1901)	D = 0.12 P = 0.01	D = 0.12–0.12	P = 0.48–0.18
*Craugastor spatulatus* (Smith, 1939)	D = 0.11 P = 0.01	D = 0.11–0.11	P = 0.08–0.18
*Craugastor yucatanensis* (Lynch, 1965)	D = 0.13 P = 0.01	D = 0.13–0.13	P = 0.07–0.94
*Duellmanohyla chamulae* (Duellman, 1961)	D = 0 P = 0.01	D = 0–0	P = 1–1
*Eleutherodactylus verrucipes* (Cope, 1885)	D = 0.14 P = 0.01	D = 0.14–0.14	P = 0.16–0.41
*Exerodonta juanitae* (Snyder, 1972)	D = 0.07 P = 0.01	D = 0.07–0.07	P = 0.1–0.16
*Exerodonta melanomma* (Taylor, 1940)	D = 0.15 P = 0.01	D = 0.15–0.15	P = 0.37–0.09
*Exerodonta sumichrasti* (Brocchi, 1879)	D = 0.1 P = 0.01	D = 0.1–0.1	P = 0.67–0.12
*Hyla euphorbiacea* (Günther, 1859)	D = 0.09 P = 0.01	D = 0.09–0.09	P = 0.26–0.62
*Incilius cristatus* (Wiegmann, 1833)	D = 0.14 P = 0.01	D = 0.14–0.14	P = 0.06–0.39
*Megastomatohyla nubicola* (Duellman, 1964)	D = 0.09 P = 0.01	D = 0.09–0.09	P = 0.3–0.93
*Plectrohyla celata* (Toal and Mendelson, 1995)	D = 0.06 P = 0.01	D = 0.06–0.06	P = 0.57–0.36
*Plectrohyla chryses* (Adler, 1965)	D = 0.03 P = 0.01	D = 0.03–0.03	P = 0.69–0.17
*Plectrohyla pentheter* (Adler, 1965)	D = 0.08 P = 0.01	D = 0.08–0.08	P = 0.61–0.33
*Plectrohyla thorectes* (Adler, 1965)	D = 0.01 P = 0.01	D = 0.01–0.01	P = 0.21–0.31
*Lithobates montezumae* (Baird, 1854)	D = 0.03 P = 0.01	D = 0.03–0.03	P = 0.35–0.81
*Lithobates dunni* (Zweifel, 1957)	D = 0.001 P = 0.01	D = 0.001–0.001	P = 0.18–0.66

(X → Z: from *L*. *catesbeianus* to endemic species; Z → X: from endemic species to *L*. *catesbeianus*) D means niche overlap (Schoener’s *D*) and P means statistical significance. Endemic species in Group 1 showed climate similarity with *L*. *catesbeianus* in two ways; in Group 2 only *L*. *catesbeianus* was significant, in Group 3 only endemic species obtain significant differences and Group 4 not show significant differences in either way.

### Climatic niche modeling

Model was constructed using a calibration with a multiplier (beta multiplier) of 3, indicating medium complexity; a Background of 10000 and a Random test of 20%. The functions used for the model were the combinations of features linear (L), quadratic (Q) and hinge (H); and redundant points were eliminated for each pixel with the "Remove Duplicates" feature. The obtained model had an AUC average of 0.91, indicating a good performance with low errors of commission and correctly identifying all locations where *L*. *catesbeianus* has been reported. The environmental variables that influenced the prediction to a greater extent were elevation, mean annual temperature and mean annual precipitation.

**Fig 3:**
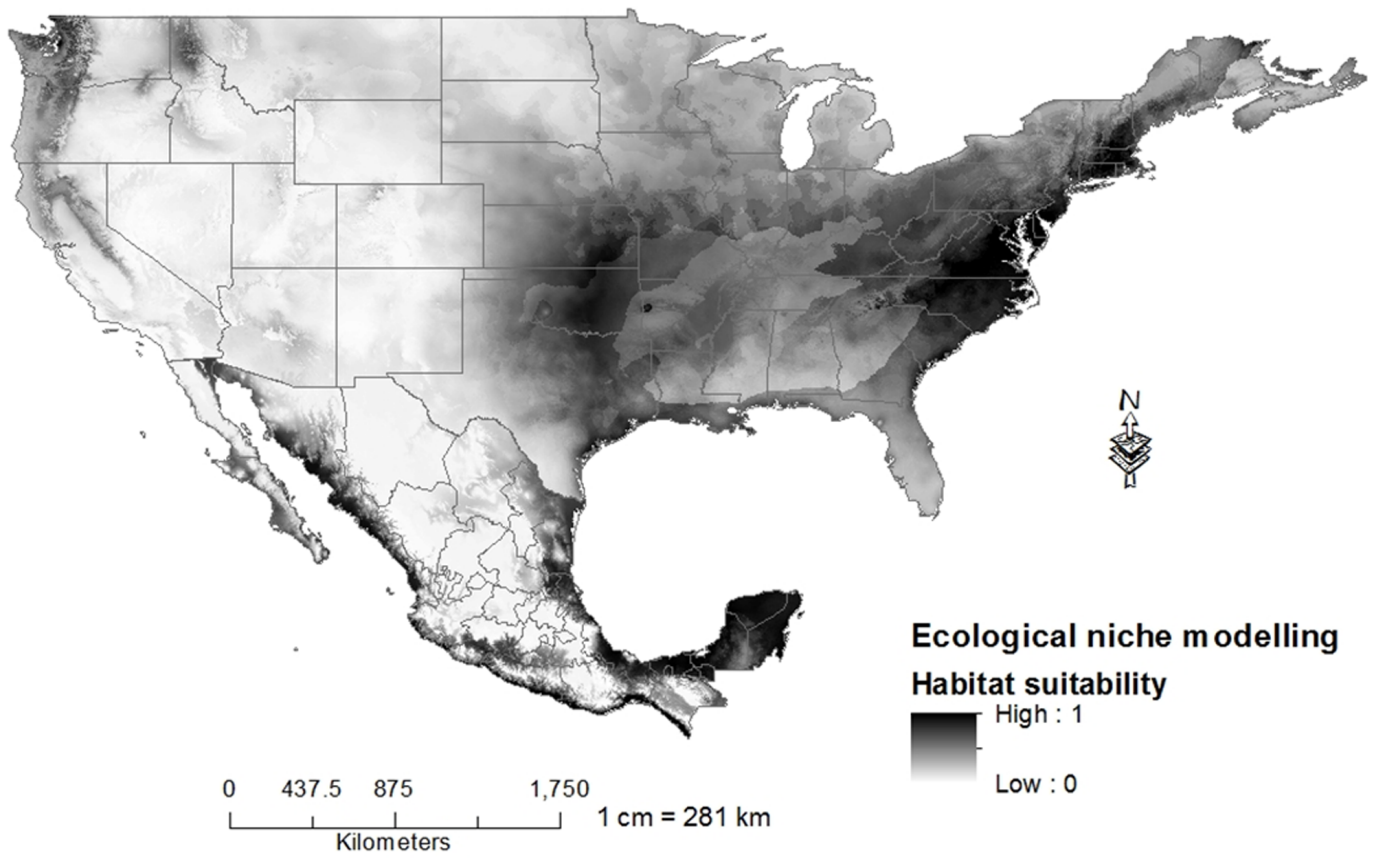
Model of habitat suitability of *L*. *catesbeianus*. Analysis under current climatic conditions. Habitat suitability is measured in an ascending scale from 0 to 1, where 0 is low habitat suitability and 1 is high suitability.

The greatest suitability of habitat in the United States out of the native range of *L*. *catesbeianus* was predicted in the states of California, Idaho, Oregon and Washington. While in Mexico, the greatest suitability of habitat for this species was predicted in ecoregions bordering the Gulf of Mexico and the Pacific Ocean. For the region bordering the Gulf of Mexico, the greatest suitability of habitat is in these ecoregions: Tropical Humid Forests, Tropical Dry Forests and Great Plains, while for the region bordering the Pacific Ocean, ecoregions of Tropical Dry Forest are those with the greatest habitat suitability. Within the ecoregion known as the deserts of North America, the Sonora Desert and Baja California had the highest habitat suitability, while for the Chihuahuan Desert the suitability is low; concentrated to a greater extent, in the central portion of this desert (Fig 3).

## Discussion

Our results obtained from equivalence test between native realized niche of bullfrog and invaded zones in Mexico, indicate that D values showed statistically significant differences. Therefore, there are no evidence to accept the null hypothesis of niche equivalency [17], enabling us to interpret that evaluated niches are ecologically distinct. Regarding similarity test, the null hypothesis proposed by Broennimann et al [46] indicates that if the observed overlap is greater than 95% of the simulated values, the entity occupies environments in both of its ranges that are more similar to each other than expected by chance. This test is strict because only considers that climate similarity will occur when statistical values are significant in both directions (native vs invaded and invaded vs native) [17]. Under these criteria, the results of the similarity test between the native niche and the invaded range by bullfrog in Mexico, allow us to reject the null hypothesis of climatic similarity between the evaluated zones.

These results provide evidence that *L*. *catesbeianus* can occupy climatically distinct niches from its native range after its introduction in a new area, supporting the hypothesis that some species can experiment changes on its niche in new environmental conditions [7, 10–12, 31, 59]. Thus, it is important to consider the dynamics of alternative niches that involve biological invasions [15, 60]. In this study, we found that during bullfrog invasion into Zones C and B, niche stability was low and niche expansion was high in relation to Zones A and D. This suggests a greater variation of realized niche in Zones C and B. These variations could imply, implicitly, changes in fundamental niche as caused by an evolutionary process [7]. However, using correlative data it is not possible to differentiate between a change caused by the evolution of physiological tolerances or a change caused by other factors such as competition or predation, among others [7, 61]. Therefore, studies at a physiological level are necessary to assess the evolutionary capacity of this species to explain the main causes that are generating variation of realized niche of bullfrog in Mexico.

Furthermore, unfilling values were high in niches of invaded Zones A, B and C. Niche unfilling represents the proportion of native niche non-overlapping with the exotic niche [15] and species that present a large amount of unfilling in the non-native range may have ample suitable habitats available in the future [16]. Under this parameter, it is possible to generate the hypothesis that the northern zone of Mexico presents an increased risk of expansion in bullfrog distribution, with respect to the southern zone of the country, due to individuals of *L*. *catesbeianus* distributed in zones C, B and A present a greater amount of available habitat to colonize. On the other hand, the climatic similarity between the regions of origin and destination is considered a basic element for successful invasions [20].

The results of climate niche overlapping between *L*. *catesbeianus* and 82 species of endemic amphibians of Mexico showed that there was climatic similarity in two ways with Group 1. Under this criterion, the habitat of Group 1 would present the greatest risk of invasion by *L*. *catesbeianus*. The climatic niche of *L*. *catesbeianus* showed significant differences when it was compared with Group 2, but these did not show significant differences from *L*. *catesbeianus*. Regarding Group 3, the analysis of similarity showed only significant differences for the endemic species, Group 4 did not present significant differences in any of the two ways. An alternate interpretation of the analysis of climatic similarity point out that when there is a significant similarity of niche for species A vs B, but not vice versa, the possible explanation is that the climatic niche environmental space of species A is more heterogeneous or potentially wider compared to species B, thus the species B cannot occur in most environmental conditions of species A. Therefore, A vs. B result in a significant similarity, while species B vs A will lead to rejection of the hypothesis [62]. Thus, it is plausible that the habitat of species classified in Group 2, as well as the habitat of species of Group 1, have a high risk of invasion by *L*. *catesbeianus*.

The environmental variables that had more of an influence in the climate niche model were elevation, annual mean temperature and annual precipitation. These results coincide with those presented in a study conducted for the potential distribution of *L*. *catesbeianus* in Brazil [18], which points out that the most influential variables present in the distribution of this species are diurnal mean temperature range, mean annual temperature and precipitation of the driest quarter. The projection of the native range of this species in Brazil shows that the areas with higher habitat suitability were the south coast and southeast of the country, and that the areas of central and northeastern Brazil can be colonized by this species. Our work coincides locating the areas of greatest suitability of habitat for *L*. *catesbeianus* in ecoregions neighboring the coast of Mexico.

The areas that showed the highest values of habitat suitability in the United States out of native range of *L*. *catesbeianus* were the states of California, Idaho, Oregon and Washington. On the other hand, the areas that showed the highest vulnerability of invasion by *L*. *catesbeianus* in Mexico were warm-humid forests, warm-dry forests and great plains bordering the Gulf of Mexico, warm-dry forests adjacent to the Pacific Ocean and the deserts of Baja California and Sonora. Contrastingly, in the Chihuahuan Desert areas of suitability were low, although *L*. *catesbeianus* is present in the states of Chihuahua [24], Durango [63] and Hidalgo [64], which would indicate that although the values of habitat suitability are low, the adaptive ability of the bullfrog to different climatic conditions is high, which indicates that the species of Groups 3 and 4 may be vulnerable to the invasion of *L*. *catesbeianus*.

In conclusion, the low niche conservatism expressed by *L*. *catesbeianus* confers it a great ability to spread and colonize new climate spaces given the capacity of its niche to shift. The analysis of niche similarity indicates that this invasive species represents a significant risk of invasion to the habitats of endemic species of frogs in Mexico, having the necessary characteristics to occupy the climatic niche of those native species. In this regard, it has been reported that the bullfrog generates negative impacts such as resource competition, direct predation or disease vector in several endemic species of amphibians in South America [65–67]. However, the effects that the invasion of this species can cause to habitats have not been evaluated in the country, and it is important to set a parameter of the damage caused by changes in native ecosystems of frog species in Mexico [25].

Also, the climate niche shifts of *L*. *catesbeianus* allows us to propose that this frog could adapt to environmental variations generated by climate change, condition that would increase its capacity for invasion if these environmental variations generate absence of competitors, predators or pathogens leading to demographic expansion and expansion of its distribution range. Several studies have tried to evaluate areas of habitat suitability of this species in different climatic scenarios projected to the future [68–69]. These models assume niche conservatism in their projections [70–71], to evaluate species response to climate change [72–73] and predict establishment and expansion of invasive species.

However, in species that do not present a static niche, it can expand, contract or change [5], reducing the predictive capacity of distribution models based on niche conservatism for species with a dynamic niche [5]. Considering that our results indicate that the bullfrog presents a dynamic niche, we suggested that it is inappropriate to evaluate the effect of climate change with models that take niche conservatism as basis. Finally, we can point out that this study constitutes a tool to take decisions on prioritizing areas for conservation of endemic frogs of Mexico when facing the risk of invasion by *L*. *catesbeianus*.

## Supporting information

S1 Fig Summary of analysis of niche overlap. Native geographic range of *L*. *catesbeianus* in the United States and invaded zones A and B in Mexico.
(DOCX)

S2 Fig Summary of analysis of niche overlap. Native geographic range of *L*. *catesbeianus* in the United States and invaded zones C and D in Mexico.
(DOCX)

S1 Table List of endemic Mexican amphibians used in this study. Each species has its number of unique records and threatened status according with IUCN, NOM-059 SEMARNAT-2010 IUCN categories: CE = critically endangered, E = endangered, V = vulnerable, NT = near threatened, LT = least concern, and DD = data deficient. NOM-059 SEMARNAT-2010: A = threatened, P = protected, Pr = special protection.
(DOCX)
