# Supplementary material for: Evidence of niche shift and invasion potential of Lithobates catesbeianus in the habitat of Mexican endemic frogs
Source: PLoS One. 2017 Sep 27;12(9):e0185086. doi: 10.1371/journal.pone.0185086 (PMC5617169; doi:10.1371/journal.pone.0185086)

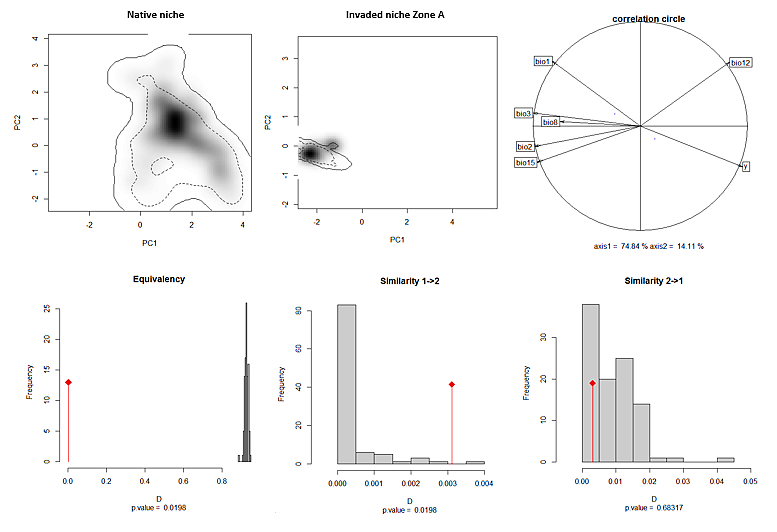
**S1 Fig. Summary of analysis of niche overlap.** Native geographic range of *L. catesbeianus* in United States and invaded zones A and B in Mexico.


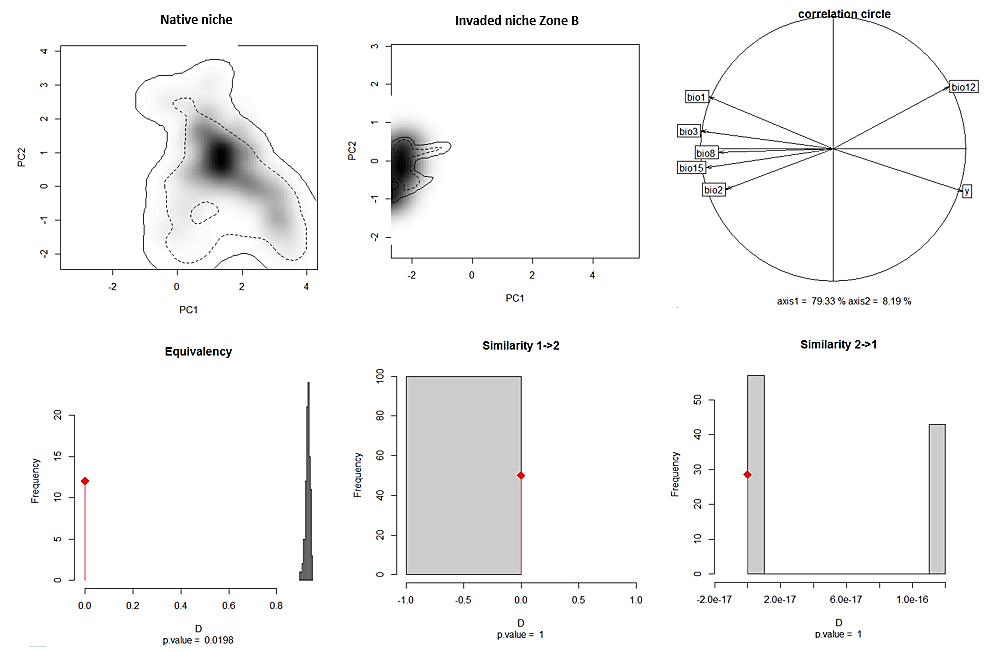

Supplement: S1 Fig — Native geographic range of L. catesbeianus in the United States and invaded zones A and B in Mexico. (DOCX) [file pone.0185086.s001.docx]
