# Supplementary material for: Evidence of niche shift and invasion potential of Lithobates catesbeianus in the habitat of Mexican endemic frogs
Source: PLoS One. 2017 Sep 27;12(9):e0185086. doi: 10.1371/journal.pone.0185086 (PMC5617169; doi:10.1371/journal.pone.0185086)
Supplement: S1 Table — Each species has its number of unique records and threatened status according with IUCN, NOM-059 SEMARNAT-2010 IUCN categories: CE = critically endangered, E = endangered, V = vulnerable, NT = near threatened, LT = least concern, and DD = data deficient. NOM-059 SEMARNAT-2010: A = threatened, P = protected, Pr = special protection. (DOCX) [file pone.0185086.s003.docx]

**S1 Table List of endemic Mexican amphibians used in this study.** Each species has its number of unique records and threatened status according with IUCN, NOM-059 SEMARNAT-2010 IUCN categories: CE=critically endangered, E=endangered, V=vulnerable, NT=near threatened, LT=least concern, and DD=data deficient. NOM-059 SEMARNAT-2010: A=threatened, P=protected, Pr=special protection.

| **Species Name** | **Number of unique points** | **IUCN** | **NOM059** |
| --- | --- | --- | --- |
| *Anaxyrus compactilis* (Wiegmann, 1833) | 126 | LC |  |
| *Anaxyrus kelloggi* (Taylor, 1938) | 221 | LC |  |
| *Anaxyrus mexicanus* (Brocchi, 1879) | 55 | NT |  |
| *Bromeliohyla dendroscarta* (Taylor, 1940) | 52 | CE |  |
| *Charadrahyla taeniopus* (Günther, 1901) | 80 | V |  |
| *Craugastor rugulosus* (Cope, *1870)* | 342 | LC |  |
| *Charadrahyla altipotens* (Duellman, 1968) | 68 | CE |  |
| *Charadrahyla chaneque*  (Duellman, 1961) | 33 | CE |  |
| *Charadrahyla nephila* (Mendelson and Campbell, 1999) | 117 | V |  |
| *Craugastor berkenbuschii* (Peters, 1870) | 75 | NT | Pr |
| *Craugastor hobartsmithi* (Taylor, 1937) | 93 | E |  |
| *Craugastor mexicanus* (Brocchi, 1877) | 488 | LC |  |
| *Craugastor montanusi* (*Lynch, 1965)* | 38 | E | Pr |
| *Craugastor occidentalis* (Taylor*, 1941)* | 83 | DD |  |
| *Craugastor omiltemanus* (Günther, 1900) | 10 | E | Pr |
| *Craugastor pozo* (Johnson and Savage, 1995) | 20 | CE |  |
| *Craugastor spatulatus* (Smith, 1939) | 6 | E | Pr |
| *Craugastor tarahumaraensis* (Taylor, 1940) | 10 | V | Pr |
| *Craugastor vocalis* (Taylor, 1940) | 181 | LC | Pr |
| *Craugastor yucatanensis* (Lynch, 1965) | 5 | NT | Pr |
| *Dendropsophus sartori (*Fitzinger, 1843) | 56 | LC |  |
| *Diaglena spatulata* (Günther, 1882) | 245 | LC |  |
| *Duellmanohyla chamulae* (Duellman, 1961) | 34 | E | Pr |
| *Duellmanohyla ignicolor* (Duellma*n, 1961)* | 82 | E | Pr |
| *Ecnomiohyla miotympanum* (Cope, 1863) | 601 | NT |  |
| *Eleutherodactylus albolabris* (Taylor 1943) | 7 |  |  |
| *Eleutherodactylus longipes* (Baird, 1859) | 37 | V |  |
| *Eleutherodactylus modestus* (Taylor, 1942) | 6 | V | Pr |
| *Eleutherodactylus nitidus* (Peters, 1870) | 178 | LC |  |
| *Eleutherodactylus nivicolimae* (Dixon and Webb, 1966) | 55 | V | Pr |
| *Eleutherodactylus teretistes* (Duellman, 1958) | 46 | DD | Pr |
| *Eleutherodactylus verrucipes* (Cope, 1885) | 9 | V | Pr |
| *Exerodonta chimalapa* (Mendelson and Campbell, 1994) | 20 | E |  |
| *Exerodonta juanitae* (Snyder, 1972) | 84 | V | A |
| *Exerodonta melanomma* (Taylor, 1940) | 99 | V | Pr |
| *Exerodonta pinorum* (Taylor, 1937) | 63 | V | Pr |
| *Exerodonta sumichrasti* (Brocchi, 1879) | 123 | LC |  |
| *Hyla euphorbiacea* (Günther, 1859) | 388 | NT |  |
| *Hyla plicata* (Brocchi, 1877) | 69 | LC | A |
| *Incilius cavifrons* (Firschein, 1950) | 22 | E |  |
| *Incilius cristatus* (Wiegmann, 1833) | 9 | CE | Pr |
| *Incilius marmoreus* (Wiegmann, 1833) | 446 | LC |  |
| *Incilius mazatlanensis* (Taylor, 1940) | 819 | LC |  |
| *Incilius occidentalis* (Camerano, 1879) | 431 | LC |  |
| *Incilius perplexus* (Taylor, 1943) | 72 | E |  |
| *Incilius spiculatus* (Mendelson, 1997) | 9 | E |  |
| *Lithobates berlandieri* (Baird, 1859) | 192 | LC |  |
| *Lithobates dunni* (Zweifel, 1957) | 33 | E | Pr |
| *Lithobates johni* (Blair, 1965) | 5 | E | P |
| *Lithobates magnaocularis* (Frost and Bagnara, 1974) | 135 | LC |  |
| *Lithobates megapoda* (Taylor, 1942) | 44 | V | Pr |
| *Lithobates montezumae* (Baird, 1854) | 49 | LC | Pr |
| *Lithobates neovolcanicus* (Hillis and Frost, 1985) | 134 | NT | A |
| *Lithobates omiltemanus* (Günther, 1900) | 9 | CE | P |
| *Lithobates pustulosus*  (Boulenger, 1883) | 91 | LC | Pr |
| *Lithobates sierramadrensis* (Taylor, 1939) | 23 | V | Pr |
| *Lithobates spectabilis* (Hillis and Frost, 1985) | 268 | LC |  |
| *Lithobates tlaloci* (Hillis and Frost, 1985) | 8 | CE | P |
| *Lithobates zweifeli* (Hillis, Frost, and Webb, 1984) | 151 | LC |  |
| *Megastomatohyla nubicola* (Duellman, 1964) | 5 | E | A |
| *Megastomatohyla pellita* (Duellman, 1968) | 28 | CE |  |
| *Pachymedusa dacnicolor* (Cope, 1864) | 429 | LC |  |
| *Plectrohyla bistincta* (Cope, 1877) | 46 | LC | Pr |
| *Plectrohyla celata* (Toal and Mendelson, 1995) | 57 | CE |  |
| *Plectrohyla cembra* (Caldwell, 1974) | 6 | CE |  |
| *Plectrohyla charadricola* (Duellman, 1964) | 7 | E | Pr |
| *Plectrohyla chryses* (Adler, 1965) | 33 | CE | Pr |
| *Plectrohyla crassa* (Brocchi, 1877) | 23 | CE | Pr |
| *Plectrohyla cyanomma* (Caldwell, 1974) | 56 | CE | Pr |
| *Plectrohyla cyclada* (Campbell and Duellman, 2000) | 5 | E |  |
| *Plectrohyla lacertosa* (Bumzahem and Smith, 1954) | 99 | E | Pr |
| *Plectrohyla mykter* (Adler and Dennis, 1972) | 7 | E | Pr |
| *Plectrohyla pentheter* (Adler, 1965) | 64 | E |  |
| *Plectrohyla robertsorum* (Taylor, 1940) | 108 | E | Pr |
| *Plectrohyla sabrina* (Caldwell, 1974) | 71 | CE | Pr |
| *Plectrohyla siopela* (Duellman, 1968) | 6 | CE |  |
| *Plectrohyla thorectes* (Adler, 1965) | 10 | CE | Pr |
| *Ptychohyla erythromma* (Taylor, 1937) | 10 | E | Pr |
| *Smilisca dentata* (Smith, 1957) | 8 | E |  |
| *Eleutherodactylus dilatus* (Davis and Dixon, 1955) | 5 | E |  |
| *Tlalocohyla godmani* (Günther, 1901) | 10 | V |  |
| *Tlalocohyla smithii* (Boulenger, 1902) | 748 | LC |  |
